# Importance of Speed of First Attempted Contact in Alzheimer’s Trial Participation

**DOI:** 10.1002/alz.088352

**Published:** 2025-01-09

**Authors:** Sarah Starling, Madeleine Sanz, Chiamaka Chukwu, John Albano, Miriam Evans, Stephanie Rutrick

**Affiliations:** ^1^ Adams Clinical, Watertown, MA USA

## Abstract

**Background:**

Identifying strategies to engage with potential participants is critical for efficient enrollment in Alzheimer’s Disease (AD) trials. Previous studies link faster speed of first contact with successful phone interview completion for Major Depressive Disorder (MDD) participants. This has not been examined in AD participants. We explored how first contact speed impacts the likelihood of AD participants completing a phone screen and how quickly that phone screen happened following initial contact.

**Method:**

Our sample includes prospective AD trial participants recruited from January to November 2023 via Facebook and Google ads. Contact information was submitted online, followed by a recruiter‐conducted phone interview. A voicemail was left for non‐responsive participants, along with an automated text and additional follow‐up calls in the following days. This analysis investigates how time lag between online submission and the first attempted recruiter call impacts completion rates of phone screens and how quickly that phone screen occurred following initial contact.

**Result:**

From January to December 2023, 6881 individuals applied to participate in AD trials through online advertisements. Time to first attempted call ranged from under an hour to 227 days, with 95.4% occurring within 72 hours. 46% of applicants eventually communicated with a recruiter. Longer delays to first call decreased the likelihood of reaching the potential participant across all calls (β = ‐.001, *p*<.001) and just those made in the first 72 hours (β = ‐.024, *p*<.001). Calls made within 24 hours yielded a 47.9% success rate, compared to 42.7% for 24‐48 hours, and 39.4% for 48‐72 hours. Unlike for MDD, time to the first attempted call wasn’t predictive of participants answering the first call (*p* = .334). It did, however, positively correlate with the number of business days between first call and eventual communication with the participant (*r* = .136, *p*<.001).

**Conclusion:**

These findings suggest that minimizing delay from a participant’s online submission to a recruiter’s first attempted call increases the likelihood of the participant completing a phone screen and shortens the time between that call and when the phone interview occurs. As fewer than half of potential participants ever spoke with a recruiter, future efforts should explore additional outreach strategies.

